# An expanded myeloid derived suppressor cell population does not play a role in gammaherpesvirus-exacerbated breast cancer metastases

**DOI:** 10.1186/1750-9378-7-22

**Published:** 2012-09-04

**Authors:** Daniel A Nelson, Vinita S Chauhan, Melanie D Tolbert, Kenneth L Bost

**Affiliations:** 1 Department of Biology, University of North Carolina at Charlotte, 9201 University City Blvd, Charlotte, North Carolina, USA

**Keywords:** Myeloid derived suppressor cells, Gammaherpesvirus, Breast cancer

## Abstract

**Background:**

Mice latently infected with murine gammaherpesvirus 68 (HV-68) and transplanted with 4 T1 breast cancer cells developed exacerbated metastatic lesions when compared to controls. The mechanisms responsible for this viral-exacerbated disease were not clear. The ability of HV-68 infection to induce S100A8 and S100A9 production and to expand a population of CD11b+Gr-1+ cells suggested that increased numbers, or activity, of viral-expanded myeloid derived suppressor cells (MDSCs) might contribute to HV-68-associated metastatic breast cancer in this model. We questioned whether mock or HV-68 infected mice with significant breast cancer might have differences in the number and/or activity of MDSCs.

**Methods:**

Myeloid-derived macrophages and dendritic cells were isolated from normal mice and cultured in vitro with HV-68 to assess S100A8 and S100A9 mRNA and protein expression. In vivo studies were performed using groups of mice that were mock treated or infected with HV-68. After viral latency was established, 4 T1 breast cancer cells were transplanted in mice. When primary breast tumors were present mice were euthanized and cells isolated for phenotyping of myeloid cell populations using FACS, and for ex vivo analysis of suppressor activity. Serum from these animals was also collected to quantify S100A8 and S100A9 levels.

**Results:**

In vitro studies demonstrated that direct exposure of myeloid cells to HV-68 did not induce increased expression of S100A8 or S100A9 mRNAs or secreted protein. HV-68 infected mice with metastatic breast cancer disease had no increases in S100A8/A9 levels and no significant increases in the numbers or activation of CD11b+Gr-1+MDSCs when compared to mock treated mice with breast cancer.

**Conclusions:**

Together these studies are consistent with the notion that expanded myeloid derived suppressor cells do not play a role in gammaherpesvirus-exacerbated breast cancer metastases. The mechanisms responsible for HV-68 induced exacerbation of metastatic breast cancer remain unclear.

## Background

Increased production of the damage-associated molecular pattern (DAMP) proteins, S100A8 and S100A9 [1], has the ability to expand myeloid derived suppressor cells (MDSCs) in vivo [2,3]. In animal models of developing cancers, increased numbers of activated MDSCs can contribute to the immune suppression and subsequent metastasis of tumor cells [4,5]. Therefore exogenous or endogenous stimuli which induce S100A8 and S100A9 during developing cancers have the potential to exacerbate such disease states by augmenting the number or activation of MDSCs [4,5].

Murine gammaherpesvirus 68 (HV-68) infection of rodents mimics the pathophysiology of Epstein Barr virus (EBV) [6,7], and makes this model a useful one for investigating EBV-associated diseases. In previous studies, we found that infection with HV-68 could induce the production of S100A8 and S100A9, and could also expand a population of CD11b+Gr-1+MDSCs in vivo [8]. This observation suggested that infection with HV-68 might exacerbate cancers if this virus could augment S100A8/A9-induced MDSCs during developing metastatic disease. In a subsequent study, we discovered that the presence of latent HV-68 exacerbated disease in a transplantable breast cancer mouse model [9]. While primary tumor growth did not vary between mock treated and HV-68 infected mice, it was clear that harboring latent virus resulted in an exacerbation of metastatic lesions in the lungs, as well as the growth of secondary tumors [9]. Theoretically, HV-68 induced expansion and activation of MDSCs could be one mechanism to explain this viral exacerbation of metastatic breast cancer.

In our previous study, the mechanisms responsible for HV-68 induced exacerbation of metastatic disease were not defined [9]. Here we questioned whether virus-expanded MDSCs might contribute to developing breast cancer. In vitro studies were performed to investigate whether direct exposure to HV-68 could induce myeloid cells to express S100A8 or S100A9. We also questioned whether HV-68 infected mice with metastatic breast cancer disease had increased S100A8/A9 levels or increased numbers of activated CD11b+Gr-1+MDSCs. We found no significant differences in virus-induced S100A8 or S100A9 or in the numbers or activation of MDSCs in infected mice bearing breast tumors. Together these studies are consistent with the notion that expanded myeloid derived suppressor cells are not responsible for gammaherpesvirus-exacerbated breast cancer metastases.

## Methods

### Animals

Six to eight week old female BALB/c mice (18–22 g) were purchased from Jackson Laboratories (Bar Harbor, ME) and housed in the vivarium in filter top cages containing sterile bedding. After arrival, mice were quarantined for at least five days, and fed chow and water ad libitum. All animal experiments were in compliance with protocols approved by the University of North Carolina at Charlotte Animal Care and Use Committee.

### Murine gammaherpesvirus-68 (HV-68)

Maintenance of viral stocks-Murine gammaherpesvirus-68 (HV-68; ATCC # VR-1465) stocks were prepared by infecting baby hamster kidney cells (BHK-21; ATCC # CCL-10) at a low multiplicity of infection (MOI), followed by preparation of cellular lysates, as described previously [9-12].

Infection of animals-Groups of mice were anesthetized with isoflurane and mock treated by intranasal instillation of saline, or infected intranasally with 6 × 10^4^ plaque forming units of HV-68. Animals were housed for 6 months following infection before transplanting 4 T1 breast cancer cells.

Assay of plaque-forming units in cell media and lysates-Replicating HV-68 was quantified by adding 1:3 serial dilutions of cell media or lysates to BALB/3 T12-3 cell (ATCC # CCL-164) monolayers. After the monolayers were incubated with virus, cells were overlayed with 1% Plaque Assay Agarose (BD Biosciences, San Diego, CA) in medium with 30% fetal bovine serum. After 5 days in 5% CO_2_, overlays were removed and cell monolayers fixed and stained with crystal violet. All serial dilutions were performed in triplicate.

### T1 breast cancer cells

Maintenance of 4 T1 cells-4 T1 cells (highly metastatic; ATCC# CRL-2539) were used as model breast cancer cells [13]. Cells were cultured in ATCC complete growth medium (RPMI 1640 medium with 10% fetal bovine serum and 2 mM L-glutamine, adjusted to contain 1.5 g/L sodium bicarbonate, 4.5 g/L glucose, 10 mM HEPES, 1.0 mM sodium pyruvate and 0.05 mM 2-mercaptoethanol).

Injection and monitoring of animals-To produce tumors, 3.5×10^4^ 4 T1 cells in 50 ml of phosphate-buffered saline (PBS) were injected into the right abdominal mammary fat pad. Following injection, animals were monitored and weighed three times a week until the last week of the experiment, when they were monitored daily.

### Bone marrow-derived macrophage and dendritic cell culture

Bone marrow derived macrophages and dendritic cells were isolated and characterized as previously described [10,14]. Cells were grown in ATCC complete growth medium supplemented with M-CSF (macrophages) or GM-CSF (dendritic cells) and incubated in 6-well plates with HV-68 for varying lengths of time, and at various HV-68 to cell ratios. For some cultures, LPS (1 ng/ml) was added as a positive control for S100A8/A9 secretion. At the indicated times, cells or culture supernatants were taken for nucleic acid and protein analysis.

### Nucleic acid analyses

Preparation of cDNA-Total RNA was isolated using Trizol (Invitrogen; Carlsbad, CA), as previously described [10,15-17]. RNA samples were incubated with RNase-free pancreatic DNase (RQ1 DNase, Promega, Madison, WI) as per the manufacturer’s instructions, the RNA precipitated with EtOH and resuspended in 50 μl of nuclease-free H_2_O. RNA concentrations were determined with a Gene Spec III spectrophotometer (Naka Instruments, Japan) using a 10 μl cuvette. For cDNA synthesis, one μg of RNA was reverse-transcribed in the presence of random hexamers (50 ng/μl), 10 mM dNTPs, 2.5 mM MgCl_2_ using ImProm-II reverse transcriptase (Promega) in the buffer supplied by the manufacturer. cDNA was precipitated with one-tenth volume of 3 M sodium acetate (pH 5.2) and 3 volumes of EtOH, and resuspended in 50 μl of nuclease-free H_2_O.

Semiquantitative PCR-mRNA transcript (cDNA) levels were examined by PCR. 100 ng of cDNA was combined with 2.5 U of Taq polymerase (Promega), 0.2 mM each dNTP**,** 25 pmol of each primer and PCR buffer containing 2.5 mM MgCl_2_ as provided by the manufacturer. Samples were cycled using 95° denaturation for 35 seconds, 60°C annealing for 75 seconds and 72°C extension for 90 seconds, with the first three cycles using extended denaturation, annealing and extension times. PCR was for 35 cycles, except for GAPDH, which was for 28 cycles. The extension time of the last cycle was for 5 min at 72°C. Forty percent of each amplified PCR product was electrophoresed on an ethidium bromide-stained 2% agarose gel and photographed under UV illumination.

PCR primer sets were designed by using IDT SciTools and purchased from IDT (Integrated DNA Technologies, Coralville, IA). Primer sets used for amplification are as follows:

**ORF65** (open reading frame-65-murid herpesvirus 4; accession no. **NC_001826**; 221 bp product).

Forward: 5’-ATG CTC CAG AAG AGG AAG GGA CAC-3’.

Reverse: 5’-TTG GCA AAG ACC CAG AAG AAG CC-3’.

**GAPDH** (glyceraldehyde-3-phosphate dehydrogenase; accession no. **NM_008084**; 346 bp-spans exons 3 to 5).

Forward: 5’-CCA TCA CCA TCT TCC AGG AGC GAG-3’.

Reverse: 5’-CAC AGT CTT CTG GGT GGC AGT GAT-3’.

**S100A8** (calgranulin A; accession no. **NM_013650**; 249 bp-spans exons 2 and 3):

Forward: 5’-GAG AAG GCC TTG AGC AAC CTC ATT G-3’.

Reverse: 5’-CCT TGT GGC TGT CTT TGT GAG ATG-3’.

**S100A9** (calgranulin B; accession no. **NM_009114**; 230 bp-spans exons 2 and 3):

Forward: 5’-GCA AGA AGA TGG CCA ACA AAG CAC-3’.

Reverse: 5’-TCA AAG CTC AGC TGA TTG TCC TGG-3’.

### Protein analyses

Western blot analysis-Protein in Laemmli sample buffer was electrophoresed on SDS-polyacrylamide gels and transferred to Immobilon P. Filters were blocked for 2 hr with 5% instant nonfat dried milk in TBS, incubated for 2 hr with primary antibody, washed for 30 min with TBS + 0.05% Tween 20, further incubated with HRP-conjugated secondary antibody for 1 hr, washed and developed with SuperSignal West Pico Chemiluminescent Substrate (Thermo Fisher, Rockford, IL). Protein bands were visualized with X-ray film. Antibodies were from R&D Systems (Minneapolis, MN).

ELISA-S100A8/S100A9 serum levels were measured using an Immundiagnostik S100A8/S100A9 (calprotectin) ELISA kit (Alpco; Salem, NH) designed to measure the concentration of the S100A8/S100A9 heterodimer.

### Fluorescence-activated cell sorting (FACS) analysis

44 days following the injection of 4 T1 tumor cells, mice were euthanized and tissues excised. Bone marrow cells were isolated from the femurs of individual mice as previously described [10,15]. Splenocyte single cell suspensions from individual mice were made by passing tissue through a 30-gauge wire mesh. Cells were washed with sterile PBS (300 × g for 10 min), resuspended in PBS containing 10% non-immune rabbit serum (Invitrogen, Camarillo, CA) and incubated on ice for 45 min with fluor-conjugated antibodies (eFluor 450-anti-CD11b and PE anti-Gr-1, eBioscience, San Diego, CA). After washing, cells were resuspended, analyzed, and then sorted using the FACSAria II cell sorter (BD Biosciences, San Diego, CA). Analyses were performed on>20,000 cells per individual spleen or bone marrow isolate.

### T-cell suppression assay

Single cell suspensions of splenocytes were prepared as described above, and red blood cells were removed using a lysing reagent (Sigma-Aldrich, St. Louis, MO). Splenic leukocytes were washed and resuspended in RPMI-1640 containing 10% fetal bovine serum. CD11b+Gr-1+ cells were then isolated using a mouse myeloid-derived suppressor cell (MDSC) isolation kit (Miltenyi Biotec, Auburn, CA).

To isolate T lymphocytes, spleens were removed from normal, uninfected mice, and splenic leukocytes prepared as described above. Total T lymphocytes were isolated using magnetic separation (Pan T cell Isolation Kit; Miltenyi Biotec).

For co-cultures, the indicated numbers of MDSC were added to 2 × 10^5^ T lymphocytes in anti-CD3 coated microtiter wells (T cell activation plates, BD Biosciences, Bedford, MA). After 72 hours of co-incubation, the amount of IFN-γ present in the culture supernatants was determined using an ELISA (DuoSet mouse IFN-γ; R&D Systems, Minneapolis, MN) as an indication of T lymphocyte activation.

### Statistics

For statistical analysis, data were analyzed using GraphPad Prism 5 software (GraphPad Software, Inc., San Diego, CA). Analyses were performed using Student’s t-test, or by one-way analysis of variance (ANOVA) with Tukey’s Multiple Comparison Test as post-test. Mean values are presented in the figures +/− the Standard Error of the Mean (SEM). Results marked with an (*) were determined to be statistically significant at P<0.05.

## Results

### HV-68 infection of cultured macrophages and dendritic cells does not increase S100A8 or S100A9 mRNA expression

**Figure 1:**
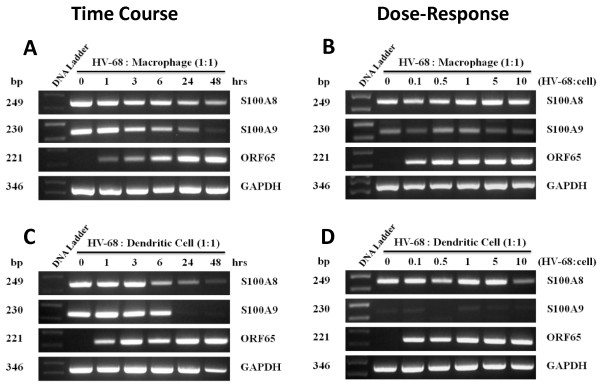
**HV-68 virus does not induce increased S100A8 and S100A9 mRNA expression in macrophages or dendritic cells in vitro.** Bone marrow-derived macrophages (Top panels **A** and **B**) or dendritic cells (Bottom panels **C** and **D**) were incubated with HV-68 virus for various lengths of time (Panels A and C-time course) or different multiplicities of infection (Panels B and D-dose-response). mRNA was isolated, treated with DNase to remove genomic DNA and used to synthesize cDNA. The time course of the viral induction of S100A8 and S100A9 mRNA was assessed by semiquantitative PCR. The viral ORF65 PCR product is presented as a proxy for viral gene expression and the PCR product from the constitutively expressed housekeeping gene GAPDH is shown to demonstrate similar amounts of cDNA in each sample. The results are presented as amplified products electrophoresed on ethidium bromide-stained agarose gels. DNA sizes in base pairs are presented to the left of the DNA standard

In previous studies, we found that infection with HV-68 could induce the production of S100A8 and S100A9, and could also expand a population of CD11b+Gr-1+MDSCs in vivo [8]. However it was not clear if these virus-induced effects were direct or indirect ones. Here we questioned whether HV-68 infection of cultured, myeloid-derived macrophages and dendritic cells could result in the increased expression of S100A8 and S100A9. Figure 1 shows S100A8 and S100A9 mRNA expression following in vitro infection of macrophages and dendritic cells with HV-68. Constitutive levels (0 hour) of S100A8 and S100A9 mRNA decreased over 48 hours in both cultured macrophages (Figure 1A) and dendritic cells (Figure 1C). Increasing the inoculum of HV-68 on these cells had no significant effect, or decreased, S100A8 and S100A9 mRNA expression in macrophages (Figure 1B) or dendritic cells (Figure 1D). The ability of HV-68 to infect macrophages and dendritic cells was demonstrated by increases in viral ORF65 mRNA expression (Figure 1). We concluded from these studies that HV-68 infection could not directly induce S100A8 or S100A9 mRNA expression in these cultured myeloid cells.

### HV-68 infection of cultured macrophages and dendritic cells does not increase the secretion of S100A8 or S100A9 protein into culture supernatants

**Figure 2:**
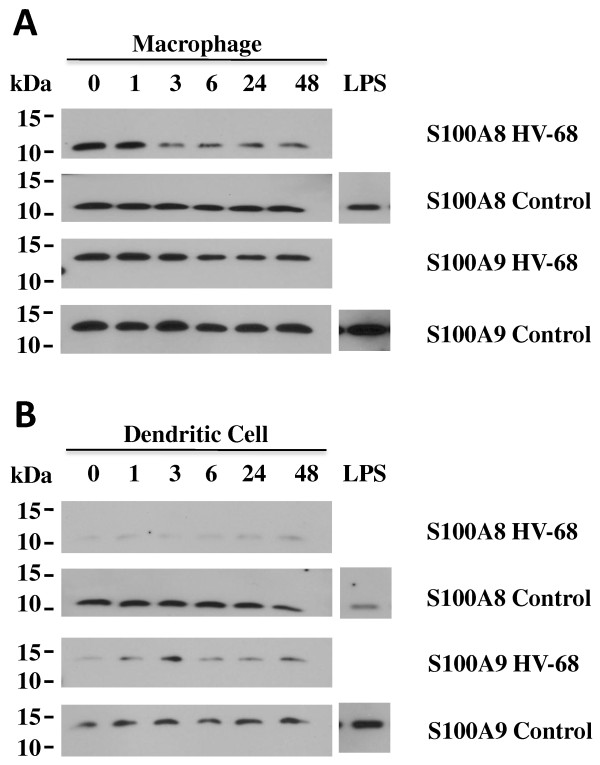
**Extracellular levels of S100A8 and S100A9 proteins do not increase after a time course of incubation of macrophages or dendritic cells with HV-68.** Bone marrow-derived macrophages (Panel **A**) or dendritic cells (Panel **B**) were incubated with HV-68 virus (MOI of 1:1) from zero to 48 hrs or with LPS (1 ng/ml). Medium was mixed with 2×SDS sample buffer, boiled and proteins electrophoresed on 15% polyacrylamide gels and protein transferred to Immobilon P probed with anti-S100A8 and anti-S100 A9 antibodies. S100A8 and S100A9 protein levels were either unchanged or reduced during the time course of the incubation. Similarly, cell lysates did not show decreased levels of intracellular S100A8 or S100A9 (data not shown)

**Figure 3:**
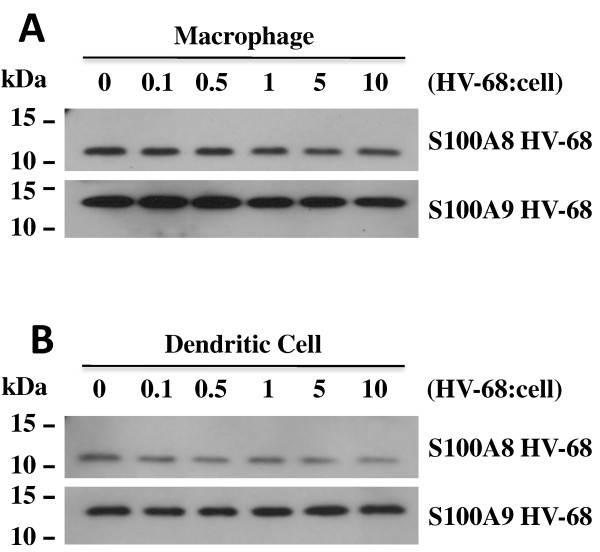
**Extracellular levels of S100A8 and S100A9 proteins do not increase in macrophages or dendritic cells with increasing doses of HV-68.** Bone marrow-derived macrophages (Panel **A**) or dendritic cells (Panel **B**) were incubated for 24 hrs with increasing amounts of HV-68 virus. Medium was mixed with 2×SDS sample buffer and protein was electrophoresed on SDS 15% polyacrylamide gels. Protein transferred to Immobilon P was probed with anti-S100A8 and S100A9 antibodies. S100A8 and S100A9 protein levels in the medium were unchanged as a result of increased amounts of HV-68 virus. Similarly, intracellular S100A8 and S100A9 levels were unchanged (data not shown)

Consistent with the mRNA data, HV-68 infection of macrophages and dendritic cells did not result in increased S100A8 or S100A9 protein secretion. Figure 2 shows that constitutive levels (0 hour) of S100A8 or S100A9 protein secreted into supernatants decreased over 48 hours in both cultured macrophages (Figure 2A) and dendritic cells (Figure 2B). Increasing the inoculum of HV-68 on these cells also decreased S100A8 and S100A9 protein secreted into supernatants in macrophages (Figure 3A) or dendritic cells (Figure 3B). We concluded from these studies that HV-68 infection could not directly induce S100A8 or S100A9 protein secretion in these cultured myeloid cells.

HV-68 infected mice harboring metastatic breast cancer have no significant increases in serum S100A8/S100A9 levels or in the numbers of CD11b+Gr-1+MDSCs ex vivo

**Figure 4:**
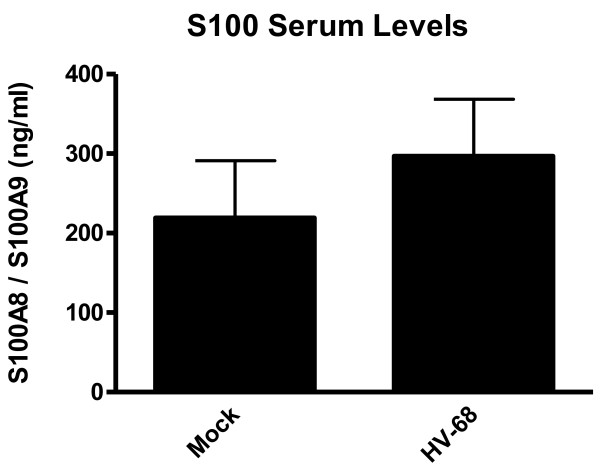
**ELISA quantification of S100A8 and S100A9 in sera of mock treated or HV-68 infected mice with metastatic breast cancer.** Groups of mice were mock treated or HV-68 infected. Six months post-infection, mice were injected with syngeneic 4 T1 mammary tumor cells into the mammary fat pad. At day 44 following tumor cell transplantation, groups of mice were euthanized and sera isolated from individual animals. Each serum sample was assayed for the presence of S100A8 and S100A9 using an ELISA capable of recognizing either protein. Results are presented as means (N = 6) ± standard errors

Previous studies demonstrated that HV-68 infection alone could induce S100A8/S100A9 in vivo during acute infection and viral latency, which was consistent with increased MDSCs in these infected mice [8]. Previous studies also demonstrated that mice with latent HV-68 infection developed exacerbated metastatic breast cancer disease [9]. To begin to address mechanisms that might be responsible for this HV-68-exacerbated cancer, we questioned whether virus-induced increases in S100A8/S100A9 levels might induce increased MDSCs in vivo. Figure 4 shows that 44 days following 4 T1 tumor cell transplantation, mock treated or HV-68 infected mice had no significant differences in serum S100A8/S100A9 levels.

**Figure 5:**
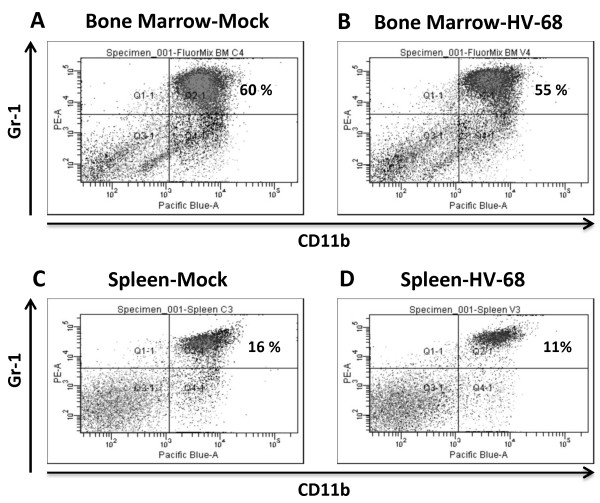
**Percentage of splenic CD11b+Gr-1+cells in mock treated and HV-68 infected mice with metastatic breast cancer.** Groups of mice were mock treated or HV-68 infected. Six months post-infection, mice were injected with syngeneic 4 T1 mammary tumor cells in the mammary fat pad. At day 44 following tumor cell transplantation, groups of mice were euthanized and tissues were isolated. Bone marrow cells and splenocytes were isolated and labeled with fluorchrome-conjugated anti-CD11b and anti-Gr-1 antibodies. FACS analysis of one representative animal per group shows the percentage of CD11b+Gr-1+cells (upper right quadrant) from the bone marrow (Panel **A**) or spleen (Panel **C**) of a mock-treated mouse, or from the bone marrow (Panel **B**) or spleen (Panel **D**) of an HV-68 infected mouse that have 4 T1 breast cancer metastases

Consistent with these results, there were no significant increases in CD11b+Gr-1+MDSCs when comparing mock treated versus HV-68 infected mice. Figure 5 shows FACS analyses from representative animals. CD11b+Gr-1+ cells from bone marrow of mock treated (Figure 5A) versus HV-68 infected (Figure 5B) mice showed no significant increases in MDSCs. Similar results were observed when analyzing splenocytes (Figure 5C versus Figure 5D).

**Figure 6:**
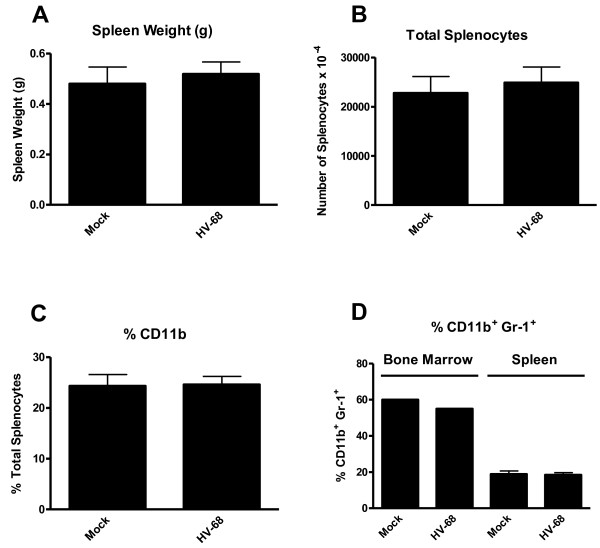
**The number and percentage of CD11b+ and CD11b+Gr-1+cells in mock treated and HV-68 infected mice with metastatic breast cancer were not significantly different.** Groups of mice were mock treated or HV-68 infected. Six months later mice were injected with syngeneic 4 T1 mammary tumor cells in the mammary fat pad. At day 44 following tumor cell transplantation, groups of mice were euthanized and tissues were isolated. Splenomegaly (Panel **A**) and the total number of leukocytes per spleen (Panel **B**) were determined. Bone marrow cells and splenic leukocytes were isolated from individual animals and labeled with fluorchrome-conjugated anti-CD11b and anti-Gr-1 antibodies. The percentage of CD11b+splenic leukocytes (Panel **C**) or CD11b+Gr-1+bone marrow cells or splenic leukocytes (Panel **D**) were determined by FACS analyses. Results are presented as mean values (N = 8) ± standard errors. These studies were performed twice with similar results

Compiling data from groups of animals mock and HV-68 infected mice with metastatic breast cancer demonstrated no significant differences in splenomegaly (Figure 6A), total splenocytes (Figure 6B), the percentage of CD11b+splenocytes (Figure 6C), or the percentage of CD11b+Gr-1+ bone marrow cells or splenocytes (Figure 6D). Clearly these studies demonstrate that HV-68 infected mice harboring metastatic breast cancer had no significant increases in the numbers of CD11b+Gr-1+ MDSCs in vivo at 44 days post-breast cancer transplantation.

### HV-68 infected mice harboring metastatic breast have no significant increases in CD11b+Gr-1+MDSCs activity in vivo

**Figure 7:**
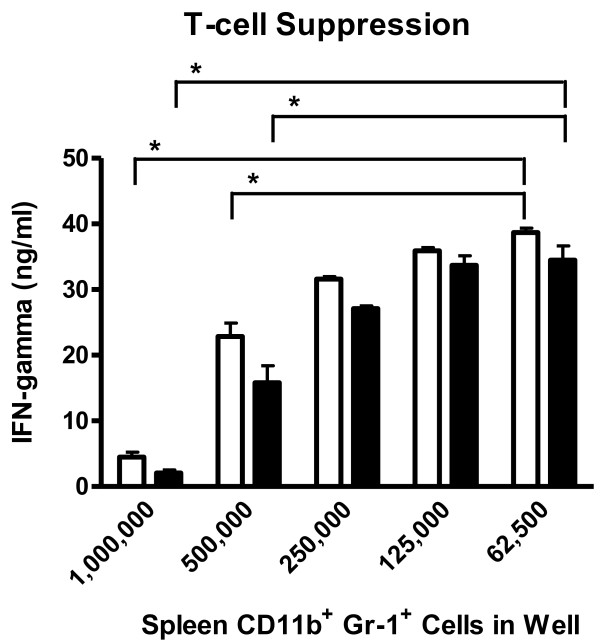
**CD11b+Gr-1+cells in mock treated and HV-68 infected mice with metastatic breast cancer suppressed anti-CD3-induced T cell activation.** Groups of mice were mock treated (white bars) or HV-68 infected (black bars). Six months post-infection, mice were injected with syngeneic 4 T1 mammary tumor cells in the mammary fat pad. At day 44 following tumor cell transplantation, groups of mice were euthanized and CD11b+Gr-1+cells were isolated from spleens of individual mice. The indicated numbers of CD11b+Gr-1+cells were added to 2 × 10^5^ cultured T lymphocytes stimulated with anti-CD3 antibodies. After 72 hours of co-incubation, culture supernatants were assayed for interferon gamma (IFN-γ) production as a measure of T cell activation. Results are presented as means (N = 4) ± standard errors with asterisks indicating a statistically significant difference (p < 0.05) as numbers of CD11b+Gr-1+cells become limiting

While the absolute numbers and percentages of MDSCs were not significantly different (Figures 5 and 6), it was possible that the suppressive activity of cells isolated from mock versus HV-68 infected mice harboring metastatic breast cancer might be different. To address this possibility, CD11b+Gr-1+ cells were isolated from individual mice and assayed for their ability to suppress anti-CD3-induced T cell activation [4]. Figure 7 shows that MDSCs isolated from either group could effectively suppress T cell induced interferon gamma secretion in co-cultures. However no significant differences in the ability to suppress T cell activation could be seen when comparing equal numbers of MDSCs isolated from mock treated (Figure 7, white bars) versus HV-68 infected (Figure 7, black bars) mice harboring metastatic breast cancer.

## Discussion

The relationship between EBV infection and breast cancer has been the subject of numerous investigations [18-22]. Some studies have demonstrated the presence of EBV genes or proteins in breast cancer biopsies [20,23-27], while other studies have suggested a relationship between aggressive breast cancers and the presence of EBV [28]. In contrast, other studies have not detected significant differences in viral gene or protein expression in breast cancer tissues [29-34]. Therefore the association of EBV with breast cancer remains controversial, and more definitive studies in patients are limited by ethical constraints.

In a recent study, we investigated the possibility that the presence of a latent gammaherpesvirus might exacerbate disease in a transplantable breast cancer using mouse models [9]. HV-68 is a murine gammaherpesvirus which mimics the pathophysiology of EBV [6,7]. Upon intranasal or oral inoculation in mice [12], there is a productive infection of epithelial cells, followed by infection of B lymphocytes, and also macrophages and dendritic cells. A marked leukocytosis (i.e. mononucleosis) and splenomegaly occurs, which peaks around 15 days post-infection and results in the establishment of latency for the life of the host. The ability of HV-68 to establish latency in mice makes this model a useful one for investigating EBV-associated diseases.

The transplantable 4 T1 mammary tumor [13] forms primary tumors at the site of injection into the mammary fat pad. Metastatic lesions begin to appear in tissues, including the lung and liver, several weeks after transplanting cells. Combining HV-68 latency, with mice developing 4 T1 mammary tumors, allowed a direct analysis of whether this gammaherpesvirus could infect breast carcinoma cells in vivo, and whether disease was exacerbated when compared to uninfected animals. Mice harboring latent HV-68 had dramatic increases in metastatic disease [9]. This exacerbated metastasis was not due to direct HV-68 infection of 4 T1 cells or to detectable levels of virus within the tumor masses. Therefore, the most likely explanation for HV-68 exacerbated metastatic breast cancer disease in this model was an indirect one. Unfortunately, the nature of this indirect effect was not clear from our previous studies, and could include factors expressed by virally infected leukocytes, or viral-induced alterations in the host’s immune response.

In the present study, we began to address possible mechanisms that might explain HV-68-exacerbated breast cancer metastasis. The rationale for the present work stems from our discovery that infection with this gammaherpesvirus resulted in an increase in S100A8/A9 production in vivo, which correlated with an increase in CD11b+Gr-1+ cells [8]. This finding was significant since this is the phenotype of MDSCs [4], and since S100A8 and S100A9 can induce expansion of MDSCs in vivo [3]. S100A8 and S100A9 are calcium-binding proteins that form noncovalent homodimers and a heterodimer (S100A8/A9) in a calcium-dependent manner [1]. These proteins have been shown to be critically important for the accumulation of MDSCs in cancer models [2,3]. MDSCs are hematopoietic precursors of macrophages, granulocytes, and dendritic cells [4]. These immature cells are CD11b+Gr-1+ and this population of cells can be dramatically expanded during some developing cancers [5]. These cells have been implicated in limiting the immune response, with a particular focus on their ability to suppress T lymphocyte activation [4]. Following HV-68 infection, we found that serum levels of S100A8 or S100A9 increase during acute infection and are maintained during the establishment of viral latency [8]. The importance of these proteins for expanding MDSCs suggested one likely mechanism to account for the accumulation of HV-68 induced CD11b+Gr1+ cells in the spleen [8].

Theoretically, if HV-68 infected mice were able to expand MDSCs during developing mammary tumor metastases, this might be one mechanism to explain viral-exacerbated disease. In vitro studies demonstrated that direct exposure of myeloid cells to HV-68 did not induce increased expression of S100A8 or S100A9 mRNAs (Figure 1) or secreted protein (Figures 2 and 3) regardless of the multiplicity of infection or time following infection. This result suggested that increased serum levels of S100A8 or S100A9 during acute infection and early latency [8] are not due to a direct effect of viral infection on macrophages or dendritic cells. Furthermore, HV-68 infected mice with metastatic breast cancer disease had no increases in serum levels of S100A8/A9 when compared to mock treated controls at 44 days post 4 T1 transplantation (Figure 4). Similarly, there were no significant differences in the percentages of CD11b+Gr-1+ cells in the spleens or bone marrow of HV-68 infected mice with a substantial tumor burden when compared to mock treated controls. Similar levels of splenomegaly, (Figure 6A), numbers of total splenocytes (Figure 6B), and percentages of total CD11b+ cells (Figure 6C) demonstrated that the absolute number of CD11b+Gr-1+ cells did not differ. It should be noted that the results shown here represent serum levels of S100A8/A9 and numbers of CD11b+Gr-1+ cells at an experimental endpoint (i.e. 44 days post 4 T1 transplantation and 226 days post-HV-68 infection). Future studies are aimed at determining if S100A8/A9 levels and CD11b+Gr-1+ numbers remained unchanged or were variable throughout the course of breast tumor development.

While the numbers of MDSCs were similar in HV-68 and mock treated mice with 4 T1 breast cancer, we also questioned whether there might be a difference in their suppressive activity. MDSCs can limit the immune response using a variety of mechanisms, and can suppress T lymphocyte function [4]. Mechanisms of MDSC suppressive activity include the production of arginase, iNOS, and reactive oxygen or nitrogen species by these cells, as well as their ability to induce T regulatory cells. Therefore we compared the suppressive activity of MDSCs isolated from HV-68 infected or mock treated mice bearing tumors using a standard co-culture suppression assay that stimulated T cells with anti-CD3 to induce interferon gamma production. While the MDSCs from both groups of animals demonstrated the ability to suppress T cell cytokine production in a dose-dependent manner, there was no significant difference in the suppressive activity of the MDSCs on a per cell basis regardless of the group of animals that these cells were isolated from (Figure 7).

Taken together these studies are consistent with the notion that expanded myeloid derived suppressor cells are not responsible for gammaherpesvirus-exacerbated breast cancer metastases. At 44 days following transplantation of 4 T1 cells, animals had a substantial primary and metastatic tumor burden [9]. Mice that harbored latent HV-68 had greatly exacerbated metastatic disease [9]. The present study does not support the notion that MDSCs contributed to exacerbated breast cancer metastases when analyzed at a time when mice had a substantial tumor burden (e.g. 44 days following transplantation). Whether there are differences in the number, or activity, of MDSCs earlier in developing breast cancer disease in HV-68 versus mock treated mice will be the subject of future investigations. Since HV-68 does not directly infect 4 T1 breast cancer cells in this model [9], the indirect mechanisms responsible for viral-induced exacerbation of metastatic breast cancer remain unclear. However the present study demonstrates the value of using HV-68 as a model to dissect possible mechanisms responsible for exacerbated disease, as such studies would not be possible using human subjects.

## Abbreviations

EBV, Epstein barr virus; HV-68, Murine gammaherpesvirus 68.

## Competing interests

The authors declare no competing interests.

## Authors’ contributions

VSC, DAN, and KLB conceived and helped design the study and participated in data collection and analysis. MET participated in data collection. All authors participated in writing and approving the final manuscript.

## References

[B1] Ehrchen JM Sunderkotter C Foell D Vogl T Roth J The endogenous toll-like receptor 4 agonist S100A8/S100A9 (calprotectin) as innate amplifier of infection, autoimmunity, and cancer J Leukoc Biol 2009 86 3 557 566 19451397 10.1189/jlb.1008647

[B2] Cheng P Corzo CA Luetteke N Yu B Nagaraj S Bui MM Ortiz M Nacken W Sorg C Vogl T Inhibition of dendritic cell differentiation and accumulation of myeloid-derived suppressor cells in cancer is regulated by S100A9 protein J Exp Med 2008 205 10 2235 2249 18809714 10.1084/jem.20080132 PMC2556797

[B3] Sinha P Okoro C Foell D Freeze HH Ostrand-Rosenberg S Srikrishna G Proinflammatory S100 proteins regulate the accumulation of myeloid-derived suppressor cells J Immunol 2008 181 7 4666 4675 18802069 10.4049/jimmunol.181.7.4666 PMC2810501

[B4] Gabrilovich DI Nagaraj S Myeloid-derived suppressor cells as regulators of the immune system Nat Rev Immunol 2009 9 3 162 174 19197294 10.1038/nri2506 PMC2828349

[B5] Tadmor T Attias D Polliack A Myeloid-derived suppressor cells-their role in haemato-oncological malignancies and other cancers and possible implications for therapy Br J Haematol 2011 153 5 557 567 21477210 10.1111/j.1365-2141.2011.08678.x

[B6] Barton E Mandal P Speck SH Pathogenesis and host control of gammaherpesviruses: lessons from the mouse Annu Rev Immunol 2011 29 351 397 21219186 10.1146/annurev-immunol-072710-081639

[B7] Rajcani J Kudelova M Murine herpesvirus pathogenesis: a model for the analysis of molecular mechanisms of human gamma herpesvirus infections Acta Microbiol Immunol Hung 2005 52 1 41 71 15957234 10.1556/AMicr.52.2005.1.2

[B8] Nelson DA Chauhan VS Tolbert MD Bost KL Murine gammaherpesvirus-68 expands, but does not activate,CD11b+ gr-1+ splenocytes in vivo J Inflamm (Lond) 2012 9 1 14 22507226 10.1186/1476-9255-9-14 PMC3431986

[B9] Chauhan VS Nelson DA Das Roy L Mukherjee P Bost KL Exacerbated metastatic disease in a mouse mammary tumor model following latent gammaherpesvirus infection Infect Agent Cancer 2012 7 1 11 22642913 10.1186/1750-9378-7-11 PMC3565933

[B10] Elsawa SF Bost KL Murine gamma-herpesvirus-68-induced IL-12 contributes to the control of latent viral burden, but also contributes to viral-mediated leukocytosis J Immunol 2004 172 1 516 524 14688362 10.4049/jimmunol.172.1.516

[B11] Gasper-Smith N Marriott I Bost KL Murine gamma-herpesvirus 68 limits naturally occurring CD4+CD25+T regulatory cell activity following infection J Immunol 2006 177 7 4670 4678 16982906 10.4049/jimmunol.177.7.4670

[B12] Peacock JW Bost KL Infection of intestinal epithelial cells and development of systemic disease following gastric instillation of murine gammaherpesvirus-68 J Gen Virol 2000 81 Pt 2 421 429 10644841 10.1099/0022-1317-81-2-421

[B13] Das Roy L Pathangey LB Tinder TL Schettini JL Gruber HE Mukherjee P Breast-cancer-associated metastasis is significantly increased in a model of autoimmune arthritis Breast Cancer Res 2009 11 4 R56 19643025 10.1186/bcr2345 PMC2750117

[B14] Gasper-Smith N Singh S Bost KL Limited IL-6 production following infection with murine gammaherpesvirus 68 Arch Virol 2006 151 7 1423 1429 16489506 10.1007/s00705-006-0725-z

[B15] Bowman CC Bost KL Cyclooxygenase-2-mediated prostaglandin E2 production in mesenteric lymph nodes and in cultured macrophages and dendritic cells after infection with salmonella J Immunol 2004 172 4 2469 2475 14764719 10.4049/jimmunol.172.4.2469

[B16] Nelson DA Marriott I Bost KL Expression of hemokinin 1 mRNA by murine dendritic cells J Neuroimmunol 2004 155 1–2 94 102 15342200 10.1016/j.jneuroim.2004.06.005

[B17] Nelson DA Tolbert MD Singh SJ Bost KL Expression of neuronal trace amine-associated receptor (Taar) mRNAs in leukocytes J Neuroimmunol 2007 192 1–2 21 30 17900709 10.1016/j.jneuroim.2007.08.006 PMC2189554

[B18] Amarante MK Watanabe MA The possible involvement of virus in breast cancer J Cancer Res Clin Oncol 2009 135 3 329 337 19009309 10.1007/s00432-008-0511-2 PMC12160138

[B19] Glaser SL Hsu JL Gulley ML Epstein-barr virus and breast cancer: state of the evidence for viral carcinogenesis Cancer Epidemiol Biomarkers Prev 2004 13 5 688 697 15159298

[B20] He JR Chen LJ Su Y Cen YL Tang LY Yu DD Chen WQ Wang SM Song EW Ren ZF Joint effects of epstein-barr virus and polymorphisms in interleukin-10 and interferon-gamma on breast cancer risk J Infect Dis 2012 205 1 64 71 22095765 10.1093/infdis/jir710

[B21] Hippocrate A Oussaief L Joab I Possible role of EBV in breast cancer and other unusually EBV-associated cancers Cancer Lett 2011 305 2 144 149 21172728 10.1016/j.canlet.2010.11.007

[B22] Joshi D Buehring GC Are viruses associated with human breast cancer? Scrutinizing the molecular evidence Breast Cancer Res Treat 2012 10.1007/s10549-011-1921-4 22274134

[B23] Arbach H Viglasky V Lefeu F Guinebretiere JM Ramirez V Bride N Boualaga N Bauchet T Peyrat JP Mathieu MC Epstein-barr virus (EBV) genome and expression in breast cancer tissue: effect of EBV infection of breast cancer cells on resistance to paclitaxel (Taxol) J Virol 2006 80 2 845 853 16378986 10.1128/JVI.80.2.845-853.2006 PMC1346837

[B24] He JR Tang LY Yu DD Su FX Song EW Lin Y Wang SM Lai GC Chen WQ Ren ZF Epstein-barr virus and breast cancer: serological study in a high-incidence area of nasopharyngeal carcinoma Cancer Lett 2011 309 2 128 136 21724319 10.1016/j.canlet.2011.05.012

[B25] Joshi D Quadri M Gangane N Joshi R Association of epstein barr virus infection (EBV) with breast cancer in rural Indian women PLoS One 2009 4 12 e8180 19997605 10.1371/journal.pone.0008180 PMC2782138

[B26] Labrecque LG Barnes DM Fentiman IS Griffin BE Epstein-barr virus in epithelial cell tumors: a breast cancer study Cancer Res 1995 55 1 39 45 7805038

[B27] Lorenzetti MA De Matteo E Gass H Martinez Vazquez P Lara J Gonzalez P Preciado MV Chabay PA Characterization of epstein barr virus latency pattern in Argentine breast carcinoma PLoS One 2010 5 10 e13603 21042577 10.1371/journal.pone.0013603 PMC2962632

[B28] Mazouni C Fina F Romain S Ouafik L Bonnier P Brandone JM Martin PM Epstein-barr virus as a marker of biological aggressiveness in breast cancer Br J Cancer 2011 104 2 332 337 21179039 10.1038/sj.bjc.6606048 PMC3031896

[B29] Baltzell K Buehring GC Krishnamurthy S Kuerer H Shen HM Sison JD Epstein-barr virus is seldom found in mammary epithelium of breast cancer tissue using in situ molecular methods Breast Cancer Res Treat 2012 132 1 267 274 22042367 10.1007/s10549-011-1841-3

[B30] Cox B Richardson A Graham P Gislefoss RE Jellum E Rollag H Breast cancer, cytomegalovirus and epstein-barr virus: a nested case-control study Br J Cancer 2010 102 11 1665 1669 20407437 10.1038/sj.bjc.6605675 PMC2883146

[B31] Murray PG Epstein-barr virus in breast cancer: artefact or aetiological agent? J Pathol 2006 209 4 427 429 16906594 10.1002/path.2032

[B32] Perkins RS Sahm K Marando C Dickson-Witmer D Pahnke GR Mitchell M Petrelli NJ Berkowitz IM Soteropoulos P Aris VM Analysis of epstein-barr virus reservoirs in paired blood and breast cancer primary biopsy specimens by real time PCR Breast Cancer Res 2006 8 6 R70 17163997 10.1186/bcr1627 PMC1797024

[B33] Perrigoue JG Den Boon JA Friedl A Newton MA Ahlquist P Sugden B Lack of association between EBV and breast carcinoma Cancer Epidemiol Biomarkers Prev 2005 14 4 809 814 15824148 10.1158/1055-9965.EPI-04-0763

[B34] Richardson AK Cox B McCredie MR Dite GS Chang JH Gertig DM Southey MC Giles GG Hopper JL Cytomegalovirus, epstein-barr virus and risk of breast cancer before age 40 years: a case-control study Br J Cancer 2004 90 11 2149 2152 15150559 10.1038/sj.bjc.6601822 PMC2409506

